# Status of the WHO recommended timing and frequency of antenatal care visits in Northern Bangladesh

**DOI:** 10.1371/journal.pone.0241185

**Published:** 2020-11-05

**Authors:** Bidhan Krishna Sarker, Musfikur Rahman, Tanjina Rahman, Tawhidur Rahman, Jubaida Jahan Khalil, Mehedi Hasan, Fariya Rahman, Anisuddin Ahmed, Dipak Kumar Mitra, Malay Kanti Mridha, Anisur Rahman

**Affiliations:** 1 Maternal and Child Health Division, icddr,b, Dhaka, Bangladesh; 2 Department of Public Health, North South University, Dhaka, Bangladesh; 3 Professor and Director of Centre of Excellence for Non-Communicable Disease, James P Grant School of Public Health, BRAC University, Dhaka, Bangladesh; University of Botswana, BOTSWANA

## Abstract

**Objective:**

There is dearth of information on the timeliness of antenatal care (ANC) uptake. This study aimed to determine the timely ANC uptake by a medically trained provider (MTP) as per the World Health Organization (WHO) recommendations and the country guideline.

**Methods:**

Cross-sectional survey was done with 2,731 women having livebirth outcome in last one year in Dinajpur, Nilphamari and Rajshahi districts, Bangladesh from August-November,2016.

**Results:**

About 82%(2,232) women received at least one ANC from a MTP. Overall, 78%(2,142) women received 4 or more ANCs by any provider and 43%(1168) from a MTP. Only 14%(378) women received their first ANC at the 1^st^ trimester by a MTP. As per 4 schedule visits by the WHO FANC model and the country guideline 8%(203) and 20%(543) women respectively received the first 2 timely ANC by a MTP; where only 1%(32) and 3%(72) received the first 3 visits timely and 0.6%(17) and 1%(29) received all the four timely visits. Factors significantly associated with the first two timely visits are: 10 or above years of schooling of women [adj. OR 2.13 (CI: 1.05, 4.30)] and their husbands [adj. OR 2.40 (CI: 1.31, 4.38)], women’s employment [adj. OR 2.32 (CI: 1.43, 3.76)], urban residential status [adj. OR 3.49 (CI: 2.46, 4.95)] and exposure to mass media [adj. OR 1.58 (CI: 1.07, 2.34)] at 95% confidence interval. According to the 2016 WHO ANC model, only 1.5%(40) women could comply with the first two ANC contacts timely by a MTP and no one could comply with all the timely 8 contacts.

**Conclusion:**

Despite high coverage of ANC utilization, timely ANC visit is low as per both the WHO recommendations and the country guideline. For better understanding, further studies on the timeliness of ANC coverage are required to design feasible intervention for improving maternal and child health.

## Introduction

Approximately, 300,000 women die annually from pregnancy or childbirth-related complications around the world and almost all of these deaths occur in low-resource settings, and most of these deaths are preventable [1, 2]. The South Asian region alone accounts for approximately one-third of the global maternal and child deaths annually [3]. The global strategy for Women’s, Children’s and Adolescents’ Health under the Sustainable Development Goal (SDG) 3 has set the targets to reduce maternal mortality ratio (MMR) to less than 70 per 100,000 live births, and the neonatal mortality to 12 per 1,000 live births or lower by 2030 [4].

High coverage of quality Antenatal Care (ANC) can play a crucial role to decrease maternal and child mortality rates and achieve national and global targets related to maternal and child health [5–7]. Studies found ANC received from skilled provider reduces the risk of pregnancy complications and adverse pregnancy outcomes such as- stillbirths, intrauterine growth retardation, preterm births, low-birth weight, fetal abnormalities and other fetal complications, possibly mediated through health promotion, disease prevention, screening and treatment which increases maternal and newborn survival [2, 8–15]. The study also emphasized on the timeliness of ANC to ensure healthy pregnancy outcomes [13].

As per the previous World Health Organization’s (WHO) recommended Focused Antenatal Care (FANC) Model; under normal circumstances, a pregnant woman should have at least four ANC visits [16]. Recently, WHO has issued “the 2016 WHO ANC model” with a new series of recommendations to improve the quality of ANC, which in turn help reducing the risk of stillbirths, complications and ensures a positive pregnancy experience. The new WHO model recommends a minimum of eight contacts. The 2016 WHO ANC Model covers 4+ ANC contacts that support the accomplishment of SDGs, which aims for reducing maternal and child mortality [6, 17, 18]. The 2016 WHO ANC model provides adequate knowledge to get prepared for birth or any complication, and lifesaving information for both mother and child as it reduces the delay of care-seeking for obstetric emergencies that contribute majority of the maternal mortality in a low-income area [7]. Though the recent 2016 WHO model recommends 8 contacts, the country guideline of Bangladesh still promotes 4 ANCs having slight time differences from the previously WHO recommended FANC model [19, 20].

Globally, the coverage of early ANC visit within 14 weeks is reported to increase from 40.9% to 58.6% from the year 1990 to 2013 [21]. However, the uptake rate differs between developed and developing countries. In the year 2013, the rate of ANC uptake in developed and developing countries was 84.8% and 48.1% respectively [21].

According to the Bangladesh Demographic and Health Surveys (BDHS) the trend of ANC coverage by a medically trained provider (MTP) is increasing [16, 22–24]. Since 2004 to 2017 (51%-82%) ANC coverage had increased by 31 point percentage [16, 22–25]. The percentage of pregnant women who made four or more ANC visit by any provider has increased from 17% in 2004 to 47% in 2017 [22–24]. In terms of the ANC coverage, geographical and regional variation exist in Bangladesh where data from BDHS 2017-’18 shows at least one ANC by a MTP is the highest in the South-west region (Khulna division91%) and the lowest in the Northeast region (Sylhet division-71%) [24]. Besides, the Northern region (Rajshahi-85% and Rangpur-75%) and the Southeast region (Chattogram-83%) also showed a higher prevalence of at least one ANC uptake. However, this regional difference fluctuated quite often since the last decade [16, 22–25].

Despite having a rise in ANC coverage in Bangladesh, it stands among the top ten countries those are contributing nearly 60% of global maternal mortality [26, 27]. Maternal and neonatal mortality remained quite unchanged in the last few years [24, 28]. Bangladesh Maternal Mortality and Health Care Survey (BMMS) shows the Maternal Mortality Ratio (MMR) is 196 per 100,000 live births in 2016 whereas it was 194 per 100,000 live births in 2010. Similar to maternal mortality, BDHS shows that neonatal deaths per 1,000 live births were 28 in 2014 and 30 in 2017–18 [16, 24, 28, 29]. Bangladesh has the highest proportion of preterm births with 19% of births occurring before gestational weeks 37 [30]. The stillbirth rate in Bangladesh is 25.4 per 1,000 births [31]. According to BMMS 2016, the major causes behind the maternal deaths are hemorrhage (31%), eclampsia (24%), abortion (7%), obstructed/prolonged labor (3%), etc. [28]. To reduce pregnancy-related complications and adverse pregnancy outcomes, timely recommended ANC is imperative.

According to the BDHS-2017-18, less than 18% women received quality ANC care. Quality care is defined as receiving four or more antenatal visits, with at least one visit from a MTP and the components include measurement of weight and blood pressure, testing of blood and urine and receipt the information on potential danger signs during pregnancy [24]. In addition to the national survey, few other studies conducted in different parts of Bangladesh that also provide information of the total number of ANC visits that a woman receives during her pregnancy period [32–34]. However, none of the surveys showed how many women secured their visits timely as per WHO recommendations as well as a country guideline. So, there is a dearth of information in Bangladesh about the timeliness of ANC visits that a pregnant woman should adhere to WHO recommendations as well as to country guideline. It is essential to look deeper into the real status of the timely ANC uptake which has a greater impact on both mother and child’s odds of survival [32, 35]. Therefore, we aimed to explore timely ANC uptake by MTPs as per the WHO recommendations and the country guideline from a cross-sectional survey in Northern Bangladesh.

## Materials and methods

### Study design and settings

It was a community-based cross-sectional study conducted in both rural and urban areas. We had two study sites in rural areas and one in urban area from 3 northern districts of Bangladesh. Rural areas were Chirirbandar, a sub-district from Dinajpur and Saidpur, a sub-district from Nilphamari in Rangpur division and the urban area was Rajshahi City Corporation from Rajshahi division. According to the Population and Housing Census, the total population of Chirirbandar was 292,500, of which 146,619 were males, and 145,881 were female [36]. For Saidpur sub-district, the total population was 264,461, of which 133,737 were males, and 130,724 were females [37]. On the other hand, for Rajshahi City Corporation the total population was 449,756, of which 232,974 were males, and 216,782 were females [38]. According to census data, we found the female literacy rate was 42% in Dinajpur, whereas for both Nilphamari and Rajshahi, it was 39% [39]. In comparison to northern divisions (Rajshahi and Rangpur), southeast (Chattogram) and northeast (Sylhet) divisions had higher Maternal Mortality Ratio (Rajshahi-173/100,000 vs Chattogram-186/100,000 and Sylhet-425/100,000), Similar to maternal mortality, under-5Child Mortality Rate of Northern region (Rajshahi-43/1,000 and Rangpur-39/1,000) is lower than Southeast (Chattogram-50/1,000) and Northeast (Sylhet-67/1,000) regions [16, 29].

### Sampling and study participants

We applied two stages cluster sampling to select study participants in the study area. We considered some socio-demographic characteristics including age, years of schooling, occupation, religion, gravida and place of residence of study participants in the sampling frame to cover a range of information on similar issues from a variety of study participants. We considered government and non-government ‘Community-Based Health Workers’ (CBHW) catchment area as a cluster. We maintained similar population coverage for cluster selection. In Chirirbandar, we considered government CBHW’s catchment area, and for Saidpur and Rajshahi, we considered non-government CBHW’s areas as our clusters. In the first stage, we randomly selected one sub-district from two rural districts each and 10 wards (lowest administrative unit of city area) from the city corporation area. Then, we randomly selected 6 clusters (CBHW’s catchment area) out of 12 in Chirirbandar of Dinajpur district, 6 clusters out of 12 clusters in Saidpur of Nilphamari district and 6 clusters from 10 clusters in Rajshahi city area.

To recruit study participants, we applied the Expanded Program of Immunization method, which is a popular spatial sampling method named as the EPI method. We selected the starting point to start data collection in the selected cluster using the EPI method. We determined the midpoint of each cluster in consultation with the community people. To ensure the randomization process in interviewing eligible participants, we spun a bottle at the midpoint of each cluster to identify the direction from where we started searching study participants [40, 41]. Interviewers visited every household on next door basis according to the direction of the bottle, and eligible participants were identified and interviewed. They collected data until the cluster’s sample size was met. During the household visit, if any eligible woman was absent, then data collectors tried at least two more times to interview her.

In each study area, around 900 women were interviewed, and finally, we completed 2731 interviews from the 3 study areas. Followings were the inclusion criteria for the study enrolment: (1) the woman had a live birth outcome in the last one year prior to interview (2) the woman passed 28 or more days after last delivery (3) the woman could hear, see and speak (4) the woman had permanent residence in the study area.

### Data collection

We conducted this survey from August to November 2016. An expert research team from the International Centre for Diarrhoeal Disease Research, Bangladesh (icddr,b) was involved in preparing a survey tool based on the research question and objectives of the study. Study investigators trained all the data collectors on the data collection tool. After that, a field test was done as pre-testing to check the feasibility of the survey tool in the real field. We checked the consistency of the survey tool and incorporated the feedback into the final version after pre-testing. The survey was administered through face-to-face interviews with eligible women.

### Data management and quality assurance

An efficient team with an experienced team leader was closely involved with the data collection for ensuring the quality data. The team leader checked the completeness of every interview on the spot after the data collection through day to day supervision. Furthermore, a project research physician (PRP) and a research investigator (RI) coordinated all the data collection teams and team leaders on a daily and weekly basis for ensuring quality data and completeness of the interview. Re-interview was done by the team leaders, PRP and RI in a significant amount to check the accuracy and validity of data. At the same time, a database template was designed by an expert programmer of the Maternal and Child Health Division (MCHD), icddr,b to enter all the data online. Dot net (Version-10) software was used for data template design as appropriate [42]. The data template was designed in such a way that while entering data, none of the variables could go missing. Skipping options were also maintained strictly and logically to avoid entry mistakes. The expert data management team entered all the data through an online database. During entering data, this team entered both pre-coded and postcoded data simultaneously. For post coding of data, the research team was also closely involved with the data management team.

### Ethics

This study had no more than minimal risk to study participants. We obtained written informed consent from each of the participants prior to the interviews. We received approval from the Institutional Review Board (IRB) of icddr,b before data collection in the field. All the participants were married, and there was no need for obtaining consent for the minors from the guardian or parents as per IRB.

### Measures

All the information given by the mothers were self-reported, however, we found 27% had a pregnancy registration card (also known as ANC card) and we checked their documents for relevance. Rest of the women who did not have a pregnancy registration card, we applied the probing technique to get the actual information from them. We determined the timely ANC coverage with regards to the WHO and the country guidelines. The primary outcome variable was the first two timely ANC visits by a skilled provider as per the WHO FANC model. Our country guideline resembles with the WHO FANC model and did not yet adopt the WHO 2016 model [43]. We considered the uptake rate of the first two timely ANC visits by MTP as per the WHO FANC guideline. We collected numbers of ANC received by a woman and when as per gestational weeks; and the providers of ANC. To estimate the ANC uptake, we considered women who had received at least one ANC from any provider. If any woman reported that, she had more than one ANC in the same week from different service providers, then ANC by the highest qualified service provider was considered. We followed the criteria of skilled or unskilled provider from the Bangladesh Demographic and Health Survey (BDHS) and considered qualified doctor, nurse/midwife/paramedic, family welfare visitor (FWV) and community skilled birth attendant (CSBA) as skilled or MTP. We used skilled provider and MTP interchangeably [16].

To analyze the timely ANC visits, we followed the criteria suggested by the two WHO models and country guideline. According to “the WHO FANC model”, the timely ANC visits refer to the 1st ANC visit between 8–12 weeks of pregnancy, the 2^nd^ ANC visit between 24–26 weeks, the 3rd ANC visit at 32^nd^ week, and the 4th ANC Visit between 36–38 weeks of gestation [43].

The timely ANC visits recommended by “the WHO 2016 ANC model”, refers to 1st contact within 12 weeks, the 2nd contact at 20^th^ week, the 3rd contact at 26^th^ week, the 4th at 30^th^ week, the 5th at 34^th^ week, the 6th at 36^th^ week, the 7th at 38^th^ week and the 8th contact at 40^th^ week [43]. Like the WHO FANC model, the country guideline also suggests at least 4 scheduled ANC visits where the timely ANC visits refer to the 1st ANC visit within 16 weeks of pregnancy, the 2^nd^ ANC visit between 24–28 weeks, the 3rd ANC visit at 32^nd^ week, and the 4th ANC Visit at 36^th^ week of gestation [20].

There is a slight difference among the 2016 WHO ANC Model, the WHO FANC Model and the Bangladesh guideline for recommending the1st timing of ANC. The 2016 WHO ANC Model recommended within 12 weeks of gestation for the 1^st^ contact whereas the WHO FANC Model recommended the 1^st^ visit between 8–12 weeks of gestation and the Bangladesh guideline recommended the 1^st^ visit within 16 weeks of gestation.

All the guidelines mentioned about the exact timing and ranges depending on gestational age. The WHO FANC model suggests timing for 1^st^, 2^nd^ and 4^th^ visits in ranges and 3^rd^ visit on the exact time of gestational age. The country guideline suggests timing for 1^st^ and 2^nd^ visits in ranges and 3^rd^ and 4^th^ visits on the exact time of gestational age. The recent WHO 2016 ANC model suggests only first contact in range of gestational weeks and remaining 7 contacts on the exact timing of gestational weeks.

We considered Anderson and Newman’s framework of health services utilization to select the covariates that are associated with ANC utilization. This framework consists of three individual determinants- i. Predisposing ii. Enabling iii. Illness level [44, 45]. We adopted age, sex as demographic and years of schooling, religion, occupation, women’s partner years of schooling and his occupation as social structure from disposing factors. In addition to previous literature and known confounder, we included these socio-demographic characteristics such as age, religion, place of residence, years of schooling status, primary occupation, number of pregnancies and living children [32, 33, 46]. Regarding age and schooling, we considered completed years. Age was categorized into three different groups such as less than or equal to 19 years, 20 to 29 years and greater than or equal to 30 years. Similarly, years of schooling was categorized into four groups as 0 to 4 years, 5 to 7 years, 8 to 9 years and greater or equivalent to 10 years of schooling. If a woman and her husband had multiple occupations, the primary occupation was considered based on their preferences in terms of their income and time spent on that occupation. We took the information about the current occupation of the survey respondents. We ensured their primary occupation by asking “what is your primary occupation?”, “What kind of work do you mainly do?”, “Are you involved in any income generating activities?” during our interview. Point to be noted here, mothers who were on maternity leave their occupation was marked as employed during the period of data collection. The women who were housewives referred to as homemakers for their occupation. By gravida, we meant the total number of confirmed pregnancies that our participant had in her lifetime.

### Statistical analysis

We performed statistical analysis using the statistical software package STATA version 13.1 [47]. To identify differences between the groups, we used the χ2 (Chi-square) test for categorical data and independent sample t-test for continuous data. We checked the linearity assumption between the predictor and the outcome variable. We found there was a non-linear relationship between the predictors and the outcome variable. Then we transformed the covariate (age and years of schooling) into categories. We estimated both unadjusted and adjusted odds ratio using simple and multiple logistic regression models considering different covariates (age, years of schooling, gravida, occupation and place of residence etc.) to see the effect of covariates on the first two timely visits by MTPs. Bivariate logistic regression analysis was conducted to examine the association between the predictor and outcome variables using the Crude Odds Ratio (COR) at a 95% confidence interval (CI). Factors that were significant with a p-value of less than 0.05 were considered for further estimation of the multiple logistic regression model. For example, the variable religion showed an insignificant relationship with the first two timely ANC visits and we excluded this variable from the regression analysis. Conventionally, p value of 0.05 is taken to indicate statistical significance. This 5% level is, however, an arbitrary minimum and p values should be much smaller to provide strong evidence. Before fitting the multiple logistic regression model, we did regression for the outcome of the first three and all four timely ANC visits by a medically trained provider, but almost all predictors were crudely insignificant for these two outcome variables separately. Furthermore, the number of observation was very low for the first three and all four timely visits by MTPs. Therefore, we considered the regression model for the first two timely visits by MTP according to the WHO FANC model.

## Results

“Table 1” describes the socio-demographic characteristics of study participants living in both urban and rural areas. Result shows that almost two-third of the respondents (62%) belonged to the age group of 20 to 29 years and the majority of the respondents were Muslims (88%). A bit more than half of women (51%) passed grade 8 and higher. Years of schooling with 10 or more were higher among the respondents in the urban area (35%) than the rural area (26%). Overall, 95% of women were homemakers. In terms of the number of pregnancies, more than one-third of women (38%) had single gravida and the 50th percentile of respondents mentioned that they had experienced two pregnancies (median 2). Almost half of women’s husband had completed 8 or more years of schooling. About three-fourth of women (77%) had television exposure and only 11% of women read newspaper or magazine.

**Table 1:** Socio-demographic status of the survey respondents by place of residence.

Traits	Rural n = 1816(%)	Urban n = 915(%)	Total, n = 2731(%)	P-value
**Age in completed years**				
≤19 years	437(24)	172(19)	609(22)	<0.01
20–29 years	1120(62)	573(63)	1693(62)	
≥30 years	259(14)	170(18)	429(16)	
**Religion**				
Islam	1540(85)	865(95)	2405(88)	<0.01
Other (Hindu, Christian)	276(15)	50(5)	326(12)	
**Years of schooling**				
0–4 years	405(22)	136(15)	541(20)	
5–7 years	565(31)	231(25)	796(29)	
8–9 years	379(21)	227(25)	606(22)	<0.01
≥10 years	467(26)	321(35)	788(29)	
**Primary occupation**				
Homemaker	1729(95)	854(93)	2583(95)	0.04
Employed*	87(5)	61(7)	148(5)	
**Number of pregnancies (Gravida)**				
1	671(37)	367(4)	1038(38)	<0.01
2	563(31)	325(36)	888(33)	
3+	582(32)	223(24)	805(29)	
**Median number of pregnancies (Gravida)**	2	2	2	
**Living children**				
1	807(44)	445(49)	1252(46)	<0.01
2	612(34)	360(39)	972(36)	
3+	397(22)	110(12)	507(19)	
**Husband’s years of schooling**				
0–4 years	636(35)	207(23)	843(31)	<0.01
5–7 years	454(25)	136(15)	590(21)	
8–9 years	307(17)	210(23)	517(19)	
≥10 years	419(23)	362(39)	781(29)	
**Husband’s primary occupation**				
Service	342(19)	265(29)	607(22)	<0.01
Business	397(22)	260(28)	657(24)	
Agriculture	355(19)	13(1)	368(13)	
Skilled labor*1	703(39)	352(39)	1055(39)	
Unemployed	19(1)	25(3)	44(2)	
**Mass media exposure (Watching TV)**				
Yes	1241(68)	860(94)	2101(77)	<0.01
No	575(32)	55(6)	630(23)	
**Mass media exposure (reading newspaper or magazine)**				
Yes	166(9)	146(16)	312(11)	<0.01
No	1650(91)	769(84)	2419(89)	

*refers Service/Business/Handicraft/Agriculture/Farm/fishing, Expatriate, and Daily Wager, etc.

*^1^ refers Handicraft, Rickshaw or Van driver, Transport worker and day labor, etc.

Table 2 presents the ANC coverage of study participants by their place of residence. Almost all the women (98%) from both rural and urban sites received at least one ANC from any provider and overall 82% women (90% urban and 77% rural women) received at least one ANC from MTP. More than three-fourths of women (78%) had 4 or more ANCs by any provider while less than half of women (43%) received 4 or more ANC by a MTP. More than half of urban women (58%) and one-third of (35%) rural women reported to have received four or more ANC from MTP. However, about 17% of women received eight or more contacts by any provider and only 4% women received eight or more contacts from MTPs. Urban women (10%) were more likely to receive 8 or more ANC by MTPs than rural women (1%).

**Table 2:** Status of ANC coverage by place of residence.

Traits	Rural n = 1816(%)	Urban n = 915(%)	Total, n = 2731(%)	P-value
**Received at least one ANC from any provider**	1770(97)	906(99)	2676(98)	<0.01
**Number of ANC visits by any provider**				
1	14(1)	0(0)	14(1)	<0.01
2	152(8)	65 (7)	217(8)	
3	214(12)	89 (10)	303(11)	
4	290 (16)	94 (10)	384 (14)	
5	351 (19)	135 (15)	486 (18)	
6	320 (17)	147 (16)	467 (17)	
7	182 (10)	166 (18)	348 (12)	
8+	247(14)	210(23)	457(17)	
Did not receive any ANC	46(3)	9(1)	55(2)	
**Received at least one ANC from MTP**	1404(77)	828(90)	2232(82)	<0.01
**Received 4+ ANC from MTPs**	636 (35)	532(58)	1168(43)	<0.01
**Received 8+ ANC from MTPs**	23(1)	95(10)	118(4)	<0.01

**Fig 1:**
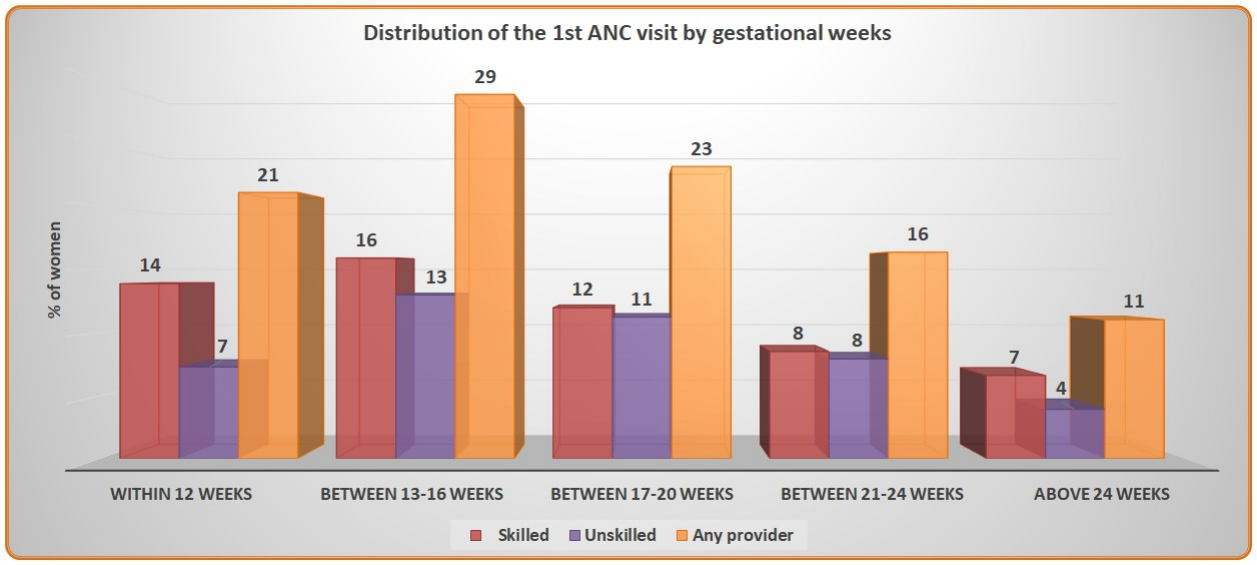
Distribution of the 1^st^ ANC visit by gestational weeks.

Fig 1 shows women received their 1^st^ ANC visit by gestational weeks from a skilled, unskilled and any provider. About one-fifth of the women (21%) received their 1st ANC within 12 weeks from any provider whereas 14% of them received from a skilled provider. The highest number of women (29%) received their 1st ANC between 13–16 weeks by any provider, whereas half of them (16%) received from a skilled provider.

“Table 3” shows almost two-thirds of the women (63%) made the timely visit 2 between 24–26 weeks from any provider followed by more than one-third of the women (35%) received the visit 4 between 36–38 weeks. Overall, only 1.2% women received all the 4 timely visits and 18% women did not receive any timely ANC visit. There was a significant difference in receiving all the timely ANC visits by any provider depending on the residence.

**Table 3:** The distribution of all 4 timely ANC visits from any provider according to the WHO FANC model by place of residence.

Trait	Rural 1770(%)	Urban 906(%)	Total 2676(%)	P-value
**Timing of 4 scheduled ANC visit discretely**				
Visit 1 (8–12 weeks)	256(14)	271(30)	527(20)	<0.01
Visit 2 (24–26 weeks)	1043(59)	636(70)	1679(63)	<0.01
Visit 3 (32 weeks)	212(12)	139(15)	351(13)	0.02
Visit 4 (36–38 weeks)	598(34)	350(39)	948(35)	0.01
**Timeliness for ANC visits**				
At least one timely visits	1407(79)	786(87)	2193(82)	<0.01
First two timely visits	142(8)	201(22)	343(13)	<0.01
First three timely visits	15(1)	41(5)	56(2)	<0.01
All four timely visits	8(0.4)	24(2.6)	32(1.2)	<0.01
Did not receive any timely visit	363(21%)	120(13%)	483(18%)	<0.01

**Fig 2:**
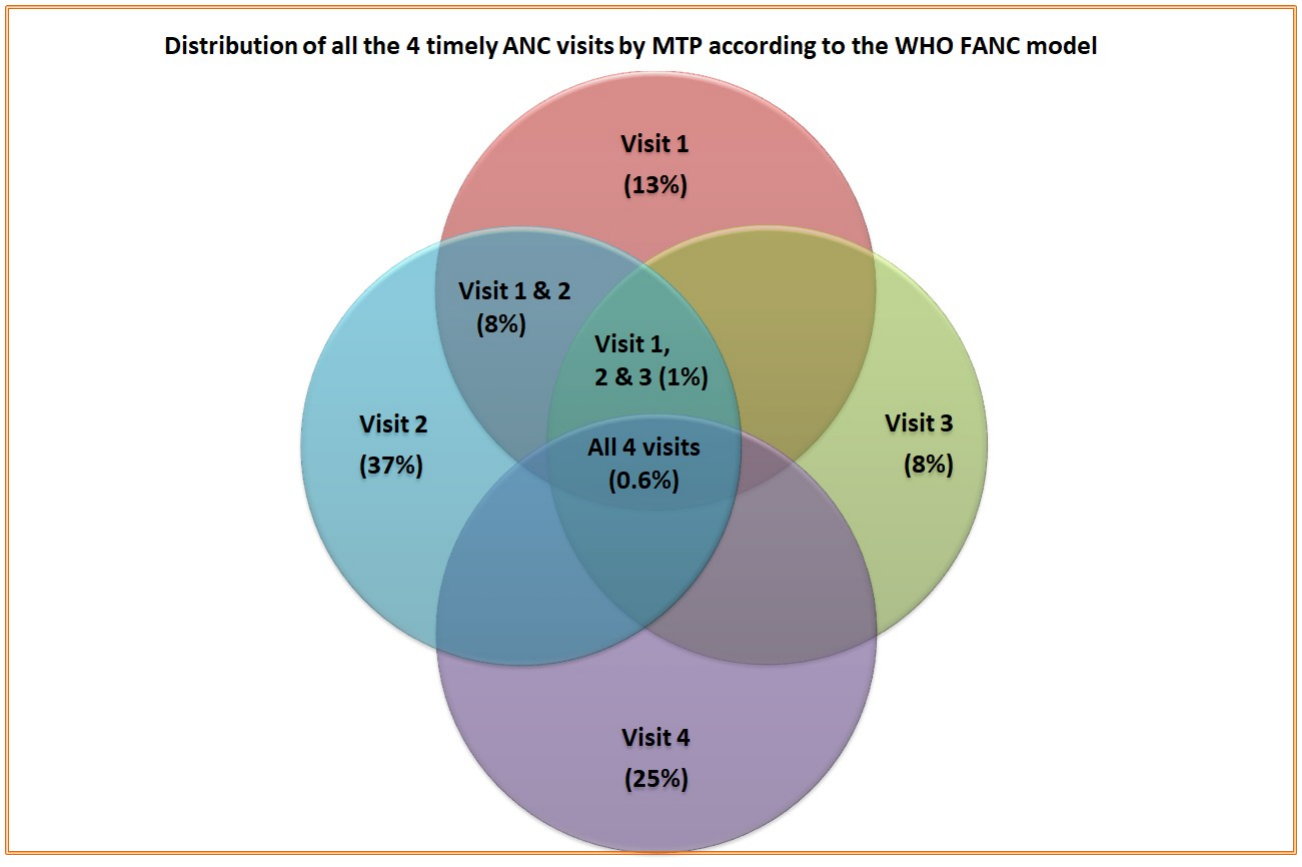
Distribution of all the 4 timely ANC visits by MTP according to the WHO FANC model.

Fig 2 shows that, only 13% women received visit 1 (between 8–12 weeks) timely, but a higher proportion of women (37%) received visit 2 (between 24–26 weeks) at the recommended time. The figure also presents that only 8% of women received the first 2 timely visits (visit 1 & 2) while less than one percent women (0.6%) received all 4 ANC visits (visit 1, 2, 3 & 4) as per recommended timing of the WHO FANC model.

“Table 4” describes that the majority women (74%) received ANC visit 2 between 24–28 weeks from any provider and 46% from a skilled provider. Half of the women received ANC visit 1 within 16 weeks from any provider while almost one-third of the women (32%) received from a skilled provider. More than one-third of the women (37%) received the first 2 timely visits (visit 1 & 2) by any provider whereas one-fifth of the women (20%) received by a skilled provider. Only 2% women received all the 4 timely visits (visit 1, 2, 3 & 4) from any provider while 1% women received from a skilled provider. In terms of all the timely ANC visits, more urban women received timely ANC visits than those of rural women. There were significant differences in receiving timely ANC visits by any provider except visit 4 and for all the timely ANC visits by a skilled provider based on place of residence.

**Table 4:** Distribution of timely ANC coverage made by the provider with place of residence according to the country guideline.

Trait	Any provider				Skilled provider			
	Rural 1770(%)	Urban 906(%)	Total 2676(%)	P-value	Rural 1770(%)	Urban 906(%)	Total 2676(%)	P-value
**Timing of 4 scheduled ANC visit discretely**								
Visit 1 (within 16 weeks)	753(43)	585(65)	1338(50)	<0.01	399(23)	446(49)	845(32)	<0.01
Visit 2 (24–28 weeks)	1251(71)	727(80)	1978(74)	<0.01	698(39)	530(59)	1228(46)	<0.01
Visit 3 (At 32 weeks)	212(12)	139(15)	351(13)	0.02	115(7)	100(11)	215(8)	<0.01
Visit 4 (At 36 weeks)	230(13)	133(15)	363(14)	0.23	151(9)	111(12)	262(10)	<0.01
**Timeliness for ANC visits**								
At least one timely visits	1544(87)	841(93)	2385(89)	<0.01	960(54)	675(75)	1635(61)	<0.01
First two timely visits	514(29)	488(54)	1002(37)	<0.01	221(13)	322(36)	543(20)	<0.01
First three timely visits	61(4)	80(9)	141(5)	<0.01	19(1)	53(6)	72(3)	<0.01
All four timely visits	21(1.2)	32(4)	53(2)	<0.01	6(0.3)	23(3)	29(1.1)	<0.01

“Table 5” presents that less than half of the women (43%) received contact 4 at 30^th^ week from any provider and near to one-third (27%) from a skilled provider. More than one-third of the women (39%) received contact 3 at 26^th^ week and contact 5 at 34^th^ week by any provider whereas one-fourth women received those contacts from a skilled provider. In line with the 2016 WHO ANC model, less than one-fifth of the women (16%) did not have any timely ANC contact from any provider and almost two-thirds of the women (63%) received at least one timing contact from a skilled provider. Only a few women (0.15%) received the timely first five contacts as per the 2016 ANC model from MTP. None of the women received all the 8 timely ANC contact either by a MTP or by any provider. Urban women were more likely to receive timely ANC contacts than those of rural women. There were significant differences in receiving timely contacts by any provider except contact 2 and 6; and by a skilled provider except contact 2 based on place of residence.

**Table 5:** The distribution of all 8 timely ANC contacts according to the 2016 WHO ANC model by provider and place of residence.

Trait	Any provider				Skilled provider			
	Rural 1770(%)	Urban 906(%)	Total 2676(%)	P-value	Rural 1770(%)	Urban 906(%)	Total 2676(%)	P-value
**ANC Timing (8 contacts)**								
Contact 1: within 12 weeks	274(15)	291(32)	565(21)	<0.01	151(9)	227(25)	378(14)	<0.01
Contact 2: 20 weeks	150(8)	61(7)	211(8)	0.14	74(4)	36(4)	110(4)	0.860
Contact 3: 26 weeks	607(34)	434(48)	1041(39)	<0.01	308(17)	306(34)	614(23)	<0.01
Contact 4: 30 weeks	659(37)	501(55)	1160(43)	<0.01	361(20)	361(40)	722(27)	<0.01
Contact 5: 34 weeks	619(35)	424(47)	1043(39)	<0.01	378(21)	320(35)	698(26)	<0.01
Contact 6: 36 weeks	230(13)	133(15)	363(14)	0.17	151(9)	111(12)	262(10)	<0.01
Contact 7: 38 weeks	260(15)	162(18)	422(16)	0.02	169(10)	127(14)	296(11)	<0.01
Contact 8: 40 weeks	130(7)	29(3)	159(6)	<0.01	93(5)	24(3)	117(4)	<0.01
**WHO ANC Timing**								
At least one timing contact	1455(82)	786(87)	2241(84)	<0.01	1006(57)	678(75)	1684(63)	<0.01
First two timing contacts	49(3)	33(4)	82(3)	0.19	19(1)	21(2)	40(1.5)	0.01
First three timing contacts	6(0.3)	15(2)	21(1)	<0.01	3(0.2)	5(0.6)	8(0.3)	0.08
First four timing contacts	4(0.2)	13(1)	17(1)	<0.01	3(0.2)	3(0.3)	6(0.2)	0.39
First five timing contacts	2(0.1)	7(1)	9(0.3)	<0.01	2(0.1)	2(0.2)	4(0.15)	0.48
First six timing contacts	-	3(0.3)	3(0.1)	0.02	-	-	-	-
First seven timing contacts	-	-	-	-	-	-	-	-
All eight timing contacts	-	-	-	-	-	-	-	-

“Table 6” shows that the first two timely ANC visits are estimated derived from the WHO FANC model. Results suggest that there is a strong association between the first two timely ANC visits and all the socio-demographic characteristics except religion.

**Table 6:** Association between the first two timely ANC visits by a skilled provider according to the WHO FANC model and socio demographic characteristics.

Traits	First two timely visits by skilled provider		P-value
	Yes n = 203 (%)	No n = 2473 (%)	
**Age in completed years**			
≤19 years	31(15.3)	570(23.1)	0.04
20–29 years	139(68.5)	1530(61.9)	
≥30 years	33(16.2)	373(15.1)	
**Religion**			
Islam	187(92.1)	2172(87.8)	0.07
Other (Hindu, Christian)	16(7.9)	301(12.2)	
**Place of residence**			
Rural	66(32.5%)	1704(68.9)	<0.01
Urban	137(67.5%)	769(31.1)	
**Years of schooling**			
0–4 years	13(6.4)	506(20.5)	<0.01
5–7 years	30(14.8)	746(30.2)	
8–9 years	43(21.2)	556(22.5)	
≥10 years	117(57.6)	665(26.9)	
**Primary occupation**			
Homemaker	170(83.7)	2361(95.5)	<0.01
Employed*	33(16.3)	112(4.5)	
**Number of pregnancies (Gravida)**			
1	95(46.8)	934(37.8)	0.02
2	63(31.0)	814(32.9)	
3+	45(22.2)	725(29.3)	
**Number of living children**			
1	115(56.7)	1124(45.4)	<0.01
2	76(37.4)	884(35.8)	
3+	12(5.9)	465(18.8)	
**Husband’s years of schooling**			
0–4 years	22(10.8)	789(31.9)	<0.01
5–7 years	25(12.3)	555(22.4)	
8–9 years	34(16.8)	479(19.4)	
≥10 years	122(60.1)	650(26.3)	
**Husband’s occupation**			
Service	79(38.9)	523(21.1)	<0.01
Business	59(29.1)	585(23.7)	
Agriculture	12(5.9)	345(13.9)	
Skilled worker	49(24.1)	981(39.7)	
Unemployed	4(2.0)	39(1.6)	
**Mass media exposure (Watching TV)**			
Yes	180(88.7)	1892(76.5)	<0.01
No	23(11.3)	581(23.5)	
**Mass media exposure (Reading Newspaper or Magazine)**			
Yes	64(31.5)	247(10.0)	<0.01
No	139(68.5)	2226(90.0)	

*refers Service/Business/Handicraft/Agriculture/Farm/fishing, and day labor, etc.

*1 refers Handicraft, Rickshaw or Van driver, Transport worker and day labor, etc.

“Table 7” shows that almost all the indicators were crudely associated with a higher prevalence of receiving the first two timely visits. After adjustment, the odds ratio of the first two timely ANC visits for the women and their husbands who had completed 10 or more years of schooling were higher than those who did not pass primary school (0–4 years of schooling). Similarly, the likelihood of receiving the first two timely visits for employed women was more than two times than the women who were homemakers. Women who had exposure to mass media (newspaper/magazine) were more likely to receive the first two timely ANC than who were not exposed. Urban women were more than three times more likely to receive the first two timely ANC from a skilled provider than those of rural women.

**Table 7:** Logistic regression of receiving the first two timely ANC visits by a skilled provider according to the WHO FANC model with the covariates.

Covariates	First two timely visits by skilled provider	
	Unadjusted OR (95% CI)	Adjusted OR (95% CI)
**Age in completed years**		
≤19 years	1.0	1.0
20–29 years	1.67(1.12, 2.49)**	1.18(0.74, 1.88)
≥ 30 years	1.62(0.98, 2.70)	1.29(0.66, 2.52)
**Place of residence**		
Rural	1.0	1.0
Urban	4.60(3.39, 6.24)***	3.49(2.46, 4.95)***
**Years of schooling**		
0-4years	1.0	1.0
5–7 years	1.56(0.81, 3.03)	1.22(0.62, 2.41)
8–9 years	3.01(1.60, 5.66)***	1.69(0.86, 3.33)
≥10 years	6.85(3.81, 12.29)***	2.13(1.05, 4.30)**
**Primary occupation**		
Homemaker	1.0	1.0
Employed*1	4.09(2.69, 6.22)***	2.32(1.43, 3.76)***
**Number of pregnancies (Gravida)**		
1	1.0	1.0
2	0.76(0.55, 1.06)	1.07(0.61, 1.86)
3+	0.61(0.42, 0.88)**	1.70(0.86, 3.36)
**Living children**		
1	1.0	1.0
2	0.84(0.62, 1.14)	0.77(0.44, 1.36)
3+	0.25(0.14, 0.46)***	0.26(0.1, 0.64)***
**Husband years of schooling**		
0–4 years	1.0	1.0
5–7 years	1.62(0.90, 2.89)	1.33(0.73, 2.43)
8–9 years	2.55(1.47, 4.40)**	1.40(0.77, 2.54)
≥10 years	6.73(4.22, 10.72)***	2.40(1.31, 4.38)***
**Husband’s primary occupation**		
Service	1.47(0.51, 4.23)	1.69(0.56, 5.13)
Business	0.98(0.33, 2.85)	1.88(0.61, 5.77)
Agriculture	0.33(0.10, 1.10)	1.46(0.41, 5.13)
Skilled labor*2	0.49(0.17, 1.42)	1.64(0.52, 5.13)
Unemployed	1.0	1.0
**Mass media exposure (Watching TV)**		
Yes	2.40(1.54, 3.75)***	0.94(0.58, 1.54)
No	1.0	1.0
**Mass media exposure (Reading Newspaper or Magazine)**		
Yes	4.15(3.0, 5.74)***	1.58(1.07, 2.34)**
No	1.0	1.0

Significant level: 0 ‘***’ 0.01 ‘**’ 0.05 ‘*’

Model adjusted for all indicators in the Table 7

CI—Confidence interval

*^1^ refers Service, Business, Handicraft, Agriculture, Farm, Fishing and Day labor, etc.

*^2^ refers Handicraft, Rickshaw or Van driver, Transport worker and Day labor, etc.

## Discussion

This study shows that almost all the women from both rural and urban sites received at least one ANC and more than three-fourth of the women received 4 or more ANC visits by any provider in their last pregnancy. Less than one-fifth of the women received 8 or more contacts by any provider whereas only 4% women received at least 8 contacts by MTPs. However, this uptake rate significantly differs between urban and rural women.

According to the WHO FANC and the 2016 ANC model, the practice of receiving the 1^st^ ANC visit within recommended time is mostly delayed. There is very little difference in terms of receiving the timely 1^st^ ANC visit by both MTPs and any provider between the two WHO models. There is slight difference (13% Vs 14% by MTP and 20% Vs 21% by any provider) between the two models on the 1st ANC uptake and that is due to different recommended timings. The FANC model recommends first ANC between 8–12 weeks while the 2016 ANC model suggests to have the first ANC contact within 12 weeks. Therefore, when ANCs are received before 8 weeks, have been considered in the 2016 ANC model and was excluded in the FANC model. However, as per country guideline, the number of the 1st ANC uptake within 16 weeks of gestation by both MTPs and any provider was two times higher than the both WHO models. As Bangladeshi country guideline recommends an elaborated time range for the initiation of ANC, it kindles curiosity among all on the difference. Similar to our findings, three more studies were done in Bangladesh suggest that the uptake of first ANC is substantially delayed and another study revealed that the reasons could be maternal age, women’s education, residence, wealth index, pregnancy intention status, child’s birth order, and wanting more children [24, 33, 48, 49]. Though no justification has found from the national guideline but our experience from working with the program in the field, we understand that culturally our women delay to disclose about their pregnancies even to their family members and relatives. They delay to seek 1^st^ ANC for few weeks thinking that pregnancy may terminate (abortion may occur) at an earlier stage, so they wait until 3 to 4 months of the pregnancy to report or visit healthcare centre. May be considering the cultural context, the national guideline adopted 1^st^ ANC by 16 weeks. Although there is no evidence, however, we are assuming that socio-cultural factors associated with delayed ANC care seeking might have been reflected on our national guideline and thus on the recommendations of the time ranges.

Though there are differences in timing, according to the WHO FANC model and the country guideline the highest proportion of the women received the timely ANC visit 2 by MTPs. Whereas according to the 2016 WHO ANC model, the highest proportion of the women complied with the contact 4 by MTPs. The country guideline mostly matches with the WHO FANC model but the 2016 WHO ANC model focused on the ANC contacts on exact weeks rather than considering ranges of weeks except ‘contact 1’ thus influences the variation of ANC uptake.

As per the WHO FANC model and the country guideline, coverage of all four timely ANC visits were extremely low and no woman could follow all the 8 timely contacts recommended by the 2016 WHO ANC model regardless of the providers. However, completing the 8 contacts is not applicable if women delivered babies before 38 weeks.

Because of the low observations for the all timely visits in both of the WHO models, the regression analysis of this study was limited to only the first two timely visits by a skilled provider as per the WHO FANC model. The regression analysis shows that women and their husbands with more years of schooling, employment, and living in the urban area were more likely to have the 1st two timely visits by MTPs.

Regarding the socio-demographic characteristics like marriage, family planning, and childbearing, Northern Bangladesh shows some variations with other Northeast and Southeast regions of Bangladesh. Early marriage (marriage before 18 years) among Northern Bangladeshi women (Rajshahi: 70% and Rangpur: 67%) is relatively higher than other parts of Bangladesh; therefore the prevalence of teenage childbearing status is also higher (Rajshahi: 33 and Rangpur: 32 vsSylhet: 14 and Chattogram: 27). However, Total Fertility Rate is lower in Northern part (Rajshahi and Rangpur: 2.1) than Northeast (Sylhet: 2.6) and Southeast (Chattogram: 2.5) regions. Regarding the modern contraceptive usage and unmet need for family planning, Rajshahi (modern method: 55%, unmet need: 10%) and Rangpur (modern method: 59%, unmet need: 8%) stand in better position than Sylhet (modern method: 45%, unmet need: 14%) and Chattogram (modern method: 45%, unmet need: 18%) divisions [24].

The BDHS 2017–18 data shows that the four or more ANC coverage raised to 47% from 31% in 2014. From the BDHS data for the Northern region, we found almost similar results with our study in terms of any ANC coverage by a MTP (BDHS: Rajshahi-84.5% and Rangpur-74.6% Vs this study-82%). The BDHS does not provide regional variation for number of ANCs, so, we couldn’t compare ANC coverage by numbers with BDHS. In addition to that, BDHS also does not present ANC coverage for 8 contacts [24]. Our study shows more ANC coverage for at least 8 ANC contacts by any provider compared to Bangladesh Multiple Indicator Cluster Survey (MICS) conducted in 2019 (17% Vs 5%) [50]. However, this difference might have induced due to having different sample size, study sites (local vs national), higher engagement of non-government organizations in providing maternal health services; especially in ANC services in our study areas, low human resource gap, access to health care, etc. [51–53].

In terms of four or more ANC coverage by any provider, a noticeable regional variation was observed for several South-Asian countries [24, 54–60]. A national survey from Afghanistan shows that in 2015 their national ANC uptake rate for four or more ANC by any provider was 18%, likewise for Bhutan- 85% in 2015, India- 51% in 2016, Myanmar- 59% in 2016, Nepal-69% in 2016 and Pakistan-51% in 2017–2018 [55, 58, 59]. Despite sharing geo-economics commonalities, these South-Asian countries exhibit a good range of variation [61].

Regarding the 1^st^ ANC uptake by gestational age, this study found that only one-fifth of the women availed their 1^st^ ANC in the 1^st^ trimester (within 12 weeks) and more than one-fourth of the women received their 1^st^ ANC during 13–16 gestational weeks by any provider. Another Bangladeshi study conducted in Netrokona district found ANC uptake by a formal provider (Doctor, midwives, nurse, FWV, CHCP, health assistant, family welfare assistant, community skilled birth attendant and NGO health workers) in the 1^st^ trimester is 18% [32]. Although the operational definition of formal provider of that study slightly differ from our definition of any provider and MTP. If we compare it with our study findings, it shows 21% of the women received the 1^st^ ANC by any provider and 14% received by MTPs [32]. We assume the difference between the definition of the skilled and unskilled care provider might have influenced the difference. Many studies conducted in different parts of Asia show, there is a huge national and regional difference in terms of the 1^st^ ANC uptake [54, 56–60]. Regarding the 1^st^ ANC uptake in the 1^st^ trimester, findings from several studies conducted in India showed the regional variation [56, 57, 60]. Indian national data showed, in 2016 more than half of the women received the 1^st^ ANC in the 1^st^ trimester, whereas in Andhra Pradesh more than three-fourth of the women and in eight other EAG states (Empowered Action Group) less than one-fifth of the women took the 1^st^ ANC uptake in the first trimester [56, 57, 60]. However, EAG states are defined as underprivileged and economically backward compared to other states of India and coverage from EAG states quite similar to our study findings [62].

Though we found Afghanistan’s four or more ANC uptake is lower than our study, but surprisingly; Afghanistan’s 1st ANC uptake in the 1st trimester was a bit higher than that of ours while Pakistan and Nepal showed more than double ANC uptake rate than our findings [54, 58, 59].

Studies show that the utilization of ANC in developing countries depends on many different factors [63–66]. Different studies done in Asian, European and African continents adopting Andersen behavioral model revealed that factors associated with underutilization of the ANC services in these regions are young age of the mothers, fewer years of schooling, lack of a paid job, poor language proficiency, support from a social network and lack of knowledge of the health care system [67]. Studies conducted in Bangladesh, different parts of India, Nepal, Afghanistan, Pakistan and Ethiopia explored that years of schooling and place of residence have influence over ANC uptake rate [32, 54, 56–60, 68–70]. In comparison to this study; findings from the above cited studies share similarity with our findings on the findings about years of schooling and place of residence. Apart from those, geographical setting and socio-economic inequalities, cultural and normative barriers are attributing to this issue [71].

Similar to other low and middle-income countries (LMIC), Bangladesh is also improving its ANC coverage. The recommended 4 ANC visits was in a view of cost-effective model and result of extensive research, further, WHO recommended 8 ANC contacts in 2016 to expedite the improvement of Maternal and Child Health related status [33, 72]. However, Bangladesh is still focusing on ensuring a higher uptake rate of 4 ANC visits with its government and non-government organizational initiatives [33, 34, 73].

Mounting all findings together from this and previous studies, we found that urban women can avail more ANC services than rural women although the ANC services are free of cost in government facilities everywhere in Bangladesh [74, 75]. Based on those shreds of evidence, it can be asserted that ANC service inequality exists based on place of residence agreeing to the fact that 78% of people living in rural Bangladesh, while 70% doctors are stationed in urban areas [76]. In addition to unavailability of skilled provider, rural Bangladeshi women face various types of challenges to access maternal health services such as: poverty, long distance of health facility, waiting time at hospital, lack of female health staff, lack of skilled birth attendant, lack of education [77–79].

Even after conducting our study in high performing areas in terms of ANC coverage, extremely low prevalence of timely ANC uptake was observed maintaining the WHO and country guidelines. We can assume further worst-case scenario for low performing areas. Although we focused to discuss about ANC uptake by skilled provider in our result mostly but we found that many other national and also global studies we discussed in our paper tend to discuss the ANC uptake by any provider and used slightly different definition of skilled provider than country guideline [32, 54–59, 68–70]. We are assuming that it is due to low ANC uptake by skilled provider in Bangladesh and other Asian and African countries. So, to understand the countrywide situation, further evidence on timely ANC uptake is required.

### Strength and limitation

Study team strictly maintained the quality of data collection in the field with close monitoring and supervision. The data derived from participants were rigorously rechecked and re-interviewed by team supervisors including a physician to minimize the scope of inaccuracy. We also checked relevant documents (such as- ANC card, pregnancy registration card, etc.) during our data collection to minimize the errors. Since this study was conducted only in part with higher ANC coverage, findings of this study hence do not represent Bangladesh uniformly. Again, the analysis was done depending on self-reported information without having a robust surveillance system; therefore, the scope of over or under-reporting may exist. Because of self-reported data, number and timing of ANC visits can be varied. According to all three guidelines, there are variations on the timing of ANC visits by exact week (e.g. 30th week) and range of weeks (e.g. 8–12 weeks); and result from range weeks will vary less but results from exact weeks may vary little bit higher. More to add, we did not explore important potential exposure variables such as household income, awareness about maternity care, cost of service, availability of healthcare services and proximity to the health facility which might have served as confounders and affected the result and the interpretation of the findings.

## Conclusions

The coverage of ANC visits is quite high but the timeliness of ANC visits is very low as per both WHO models and country guideline. Initiation for the first ANC visit is also highly delayed. Government and non-government maternal health programs should focus on ensuring timely ANC visits. Ensuring at least 4 timely visits may help to make a way forward for Bangladesh endorsing 8 ANC contacts in the near future that is recommended by the recent 2016 ANC model. We suggest policy makers to promote education, women’s employment and health education through mass media as well as to ensure universal maternal healthcare coverage. Understanding the significance of timely ANC visits we further suggest to carry out more parallel studies both in countrywide and regional perspective putting emphasis on the feasibility of 8 contacts. The findings of this study will help the program and policy makers to design interventions to improve antenatal care coverage maintaining timeliness and thus reduce maternal and child mortality across Bangladesh.

## Supporting information

S1 File (DOCX)

S1 Data (DTA)

## References

[1] Alkema L, Chou D, Hogan D, Zhang S, Moller A-B, Gemmill A, et al Global, regional, and national levels and trends in maternal mortality between 1990 and 2015, with scenario-based projections to 2030: a systematic analysis by the UN Maternal Mortality Estimation Inter-Agency Group. The Lancet. 2016;387(10017):462–74. 10.1016/S0140-6736(15)00838-7 PMC5515236 26584737

[2] Tunçalp Ӧ, Pena-Rosas JP, Lawrie T, Bucagu M, Oladapo OT, Portela A, et al WHO recommendations on antenatal care for a positive pregnancy experience-going beyond survival. BJOG. 2017;124(6):860–2. 28190290 10.1111/1471-0528.14599

[3] Victora CG, Requejo JH, Barros AJ, Berman P, Bhutta Z, Boerma T, et al Countdown to 2015: a decade of tracking progress for maternal, newborn, and child survival. The Lancet. 2016;387(10032):2049–59. 10.1016/S0140-6736(15)00519-X PMC7613171 26477328

[4] General Assembly UN. Global indicator framework for the Sustainable Development Goals and targets of the 2030 Agenda for Sustainable Development 2017 [https://unstats.un.org/sdgs/indicators/Global%20Indicator%20Framework_A.RES.71.313%20Annex.pdf.

[5] Phommachanh S, Essink DR, Jansen M, Broerse JE, Wright P, Mayxay M. Improvement of Quality of Antenatal Care (ANC) Service Provision at the Public Health Facilities in Lao PDR: Perspective and Experiences of Supply and Demand Sides. BMC pregnancy and childbirth. 2019;19(1):255 10.1186/s12884-019-2345-0 31331276 PMC6647136

[6] Benova L, Tunçalp Ö, Moran AC, Campbell OMR. Not just a number: examining coverage and content of antenatal care in low-income and middle-income countries. BMJ global health. 2018;3(2):e000779 10.1136/bmjgh-2018-000779 29662698 PMC5898334

[7] Thaddeus S, Maine D. Too far to walk: maternal mortality in context. Social science & medicine. 1994;38(8):1091–110. 10.1016/0277-9536(94)90226-7 8042057

[8] Ahmed Z, Khoja S, Tirmizi SS. Antenatal care and the occurrence of Low Birth Weight delivery among women in remote mountainous region of Chitral, Pakistan. 2012.

[9] Kadapatti MG, Vijayalaxmi A. Antenatal Care the Essence of New Born Weight and Infant Development. International Journal of Scientific and Research Publication. 2012;2(3).

[10] Uddin Mian N, Alvi MA, Malik MZ, Iqbal S, Zakar R, Zakar MZ, et al Approaches towards improving the quality of maternal and newborn health services in South Asia: challenges and opportunities for healthcare systems. Globalization and health. 2018;14(1):1–8. 29409528 10.1186/s12992-018-0338-9 PMC5802097

[11] Hollowell J, Oakley L, Kurinczuk JJ, Brocklehurst P, Gray R. The effectiveness of antenatal care programmes to reduce infant mortality and preterm birth in socially disadvantaged and vulnerable women in high-income countries: a systematic review. BMC pregnancy and childbirth. 2011;11(1):13 10.1186/1471-2393-11-13 21314944 PMC3050773

[12] Darmstadt GL, Bhutta ZA, Cousens S, Adam T, Walker N, De Bernis L, et al Evidence-based, cost-effective interventions: how many newborn babies can we save? The Lancet. 2005;365(9463):977–88. 10.1016/S0140-6736(05)71088-6 15767001

[13] Lucas AO, Stoll BJ, Bale JR. Improving birth outcomes: meeting the challenge in the developing world: National Academies Press; 2003. 25057689

[14] Debiec KE, Paul KJ, Mitchell CM, Hitti JE. Inadequate prenatal care and risk of preterm delivery among adolescents: a retrospective study over 10 years. American journal of obstetrics and gynecology. 2010;203(2):122.e1–e6. 10.1016/j.ajog.2010.03.001 20471628

[15] Beeckman K, Louckx F, Downe S, Putman K. The relationship between antenatal care and preterm birth: the importance of content of care. The European Journal of Public Health. 2013;23(3):366–71. 10.1093/eurpub/cks123 22975393

[16] National Institute of Population Research and Training (NIPORT), Mitra and Associates, ICF International. Bangladesh Demographic and Health Survey 2014. NIPORT, MEASURE Evaluation, and icddr, b Dhaka, Bangladesh; 2016.

[17] WHO, UNICEF, Mathers C. Global strategy for women’s, children’s and adolescents’ health (2016–2030). Organization. 2016. 10.2471/BLT.16.174714 PMC4850547 27147756

[18] World Health Organization. World health statistics 2016: monitoring health for the SDGs sustainable development goals: World Health Organization; 2016.

[19] Ministry of Health and Family Welfare (MOHFW). Health, Population, Nutrition eToolkit for Field Workers. 2018.

[20] Ministry of Health and Family Welfare (MOHFW). Union Health and Family welfare Center operating manual. 2014.

[21] Moller AB, Petzold M, Chou D, Say L. Early antenatal care visit: a systematic analysis of regional and global levels and trends of coverage from 1990 to 2013. The Lancet Global Health. 2017;5(10):e977–e83. 10.1016/S2214-109X(17)30325-X 28911763 PMC5603717

[22] National Institute of Population Research and Training (NIPORT), Mitra and Associates, ORC Macro. Bangladesh Demographic and Health Survey 2004. NIPORT, MEASURE Evaluation, and icddr, b Dhaka, Bangladesh; 2005.

[23] National Institute of Population Research and Training (NIPORT), Mitra and Associates, ICF International. Bangladesh Demographic and Health Survey 2011. NIPORT, MEASURE Evaluation, and icddr, b Dhaka, Bangladesh; 2013.

[24] National Institute of Population Research and Training (NIPORT), ICF. Bangladesh Demographic and Health Survey 2017–18: Key Indicators. NIPORT, MEASURE Evaluation, and icddr, b Dhaka, Bangladesh; 2019.

[25] National Institute of Population Research and Training (NIPORT), Mitra and Associates, Macro International. Bangladesh Demographic and Health Survey 2007. NIPORT, MEASURE Evaluation, and icddr, b Dhaka, Bangladesh; 2009.

[26] Moinuddin M, Christou A, Hoque DME, Tahsina T, Salam SS, Billah SM, et al Birth preparedness and complication readiness (BPCR) among pregnant women in hard-to-reach areas in Bangladesh. PLoS One. 2017;12(12). 10.1371/journal.pone.0189365 29228050 PMC5724858

[27] World Health Organization, Unicef. Trends in maternal Mortality: 1990–2015: Estimates from WHO, UNICEF, UNFPA, World Bank Group and the United Nations Population Division. 2015.

[28] National Institute of Population ResearchTraining, International Centre for Diarrhoeal Disease Research Bangladesh, MEASURE Evaluation. Bangladesh Maternal Mortality and Health Care Survey 2016: Preliminary Report. 2017.

[29] National Institute of Population Research Training, MEASURE Evaluation, ICDDR B. Bangladesh maternal mortality and health care survey 2010. NIPORT, MEASURE Evaluation, and icddr, b Dhaka, Bangladesh; 2012.

[30] Rahman A, Rahman M, Pervin J, Razzaque A, Aktar S, Ahmed JU, et al Time trends and sociodemographic determinants of preterm births in pregnancy cohorts in Matlab, Bangladesh, 1990–2014. BMJ global health. 2019;4(4):e001462 10.1136/bmjgh-2019-001462 31423346 PMC6688682

[31] Halim A, Aminu M, Dewez JE, Biswas A, Rahman AF, van den Broek N. Stillbirth surveillance and review in rural districts in Bangladesh. BMC pregnancy and childbirth. 2018;18(1):224 10.1186/s12884-018-1866-2 29914393 PMC6004696

[32] Siddique AB, Perkins J, Mazumder T, Haider MR, Banik G, Tahsina T, et al Antenatal care in rural Bangladesh: gaps in adequate coverage and content. PLoS One. 2018;13(11). 10.1371/journal.pone.0205149 30452444 PMC6242304

[33] Islam MM, Masud MS. Determinants of frequency and contents of antenatal care visits in Bangladesh: Assessing the extent of compliance with the WHO recommendations. PLoS One. 2018;13(9). 10.1371/journal.pone.0204752 PMC6160162 30261046

[34] Mohammad KA, Zahura FT, Rahman MM. Importance of maternal education on antenatal care visits in Bangladesh. Bangladesh Journal of Scientific Research. 2017;30(1–2):23–33.

[35] Souza JP, Tunçalp Ö, Vogel J, Bohren M, Widmer M, Oladapo O, et al Obstetric transition: the pathway towards ending preventable maternal deaths. BJOG: An International Journal of Obstetrics & Gynaecology. 2014;121:1–4. 10.1111/1471-0528.12735 24641529

[36] Bangladesh Bureau of Statistics (BBS), Statistics and Informatics Division, Ministry of Planning. Bangladesh Population and Housing Census 2011, Community report, Dinajpur. Bangladesh Bureau of Statistics (BBS), Statistics and Informatics Division (SID), Ministry of Planning; 2014.

[37] Bangladesh Bureau of Statistics, Statistics and Informatics Division, Ministry of Planning. Population and Housing Census, Community report, Nilphamari. Bangladesh Bureau of Statistics (BBS), Statistics and Informatics Division (SID), Ministry of Planning; 2013.

[38] Bangladesh Bureau of Statistics (BBS), Statistics and Informatics Division, Ministry of Planning. District Statistics 2011, Rajshahi. Bangladesh Bureau of Statistics (BBS), Statistics and Informatics Division (SID), Ministry of Planning; 2013.

[39] Bangladesh Bureau of Statistics, Statistics and Informatics Division, Ministry of Planning. Population and Housing Census 2011. Bangladesh Bureau of Statistics (BBS), Statistics and Informatics Division (SID) and Ministry of Planning; 2015.

[40] Bostoen K, Chalabi Z. Optimization of household survey sampling without sample frames. International journal of epidemiology. 2006;35(3):751–5. 10.1093/ije/dyl019 16481364

[41] Myatt M. A short guide to undertaking surveys using the Simple Spatial Survey Method (S3M)2012.

[42] Dot net framework [https://dotnet.microsoft.com/download/dotnet-framework.

[43] World Health Organization. WHO recommendations on antenatal care for a positive pregnancy experience: World Health Organization; 2016. 28079998

[44] Tesfaye G, Chojenta C, Smith R, Loxton D. Application of the Andersen-Newman model of health care utilization to understand antenatal care use in Kersa District, Eastern Ethiopia. PLoS One. 2018;13(12). 10.1371/journal.pone.0208729 30521640 PMC6283597

[45] Andersen R, Newman JF. Societal and individual determinants of medical care utilization in the United States. The Milbank Memorial Fund Quarterly Health and Society. 1973:95–124. 4198894

[46] Okedo-Alex IN, Akamike IC, Ezeanosike OB, Uneke CJ. Determinants of antenatal care utilisation in sub-Saharan Africa: a systematic review. BMJ open. 2019;9(10):e031890 10.1136/bmjopen-2019-031890 31594900 PMC6797296

[47] StataCorp. Stata Statistical Software: Release 13. College Station, TX StataCorp LP; 2013.

[48] Jo Y, Alland K, Ali H, Mehra S, LeFevre AE, Pak SE, et al Antenatal care in rural Bangladesh: current state of costs, content and recommendations for effective service delivery. BMC Health Serv Res. 2019;19(1):861 10.1186/s12913-019-4696-7 31752841 PMC6869180

[49] Kamal SM, Hassan CH, Islam MN. Factors associated with the timing of antenatal care seeking in Bangladesh. Asia Pacific Journal of Public Health. 2015;27(2):NP1467–NP80. 10.1177/1010539513485786 24097925

[50] Bangladesh Bureau of Statistics (BBS). Bangladesh Multiple Indicator Cluster Survey 2019, Key Findings. Bangladesh Bureau of Statistics (BBS); 2019.

[51] Bangladesh Bureau of Statistics (BBS), Statistics and Informatics Division, Ministry of Planning. Bangladesh Population and Housing Census 2011. Bangladesh Bureau of Statistics (BBS), Statistics and Informatics Division, Ministry of Planning; 2015.

[52] Human Resource management (HRM), Ministry of Health and Family Welfare (MOHFW). HRH Data Sheet 2014. Human Resource management (HRM), Ministry of Health and Family Welfare (MOHFW), Bangladesh Secretariat, Dhaka; 2014.

[53] World Mission Prayer League (LAMB Hospital). LAMB Annual Report 2017. [https://static1.squarespace.com/static/5c72ccf37a1fbd5b042f922c/t/5ca4bebe4e17b61118e6b70f/1554300705648/LAMB+Annual+Report+2017.pdf.

[54] Central Statistics Organization (CSO), Ministry of Public Health (MoPH), ICF. Afghanistan Demographic and Health Survey 2015. Central Statistics Organization (CSO), Islamabad, Pakistan, and Rockville, Maryland, USA: NIPS and ICF; 2017.

[55] United Nations International Children’s Emergency Fund (unicef). Antenatal care 2016 [https://data.unicef.org/topic/maternal-health/antenatal-care/.

[56] Gupta R, Talukdar B. Frequency and Timing of Antenatal Care Visits and its Impact on Neonatal Mortality in EAG States of India. Journal of Neonatal Biology. 2017;6(3):263.

[57] International Institute for Population Sciences (IIPS), ICF. India National Family Health Survey NFHS‐4 2015–16. Mumbai: IIPS; 2017.

[58] Ministry of Health, Nepal, New ERA, ICF. Nepal Demographic and Health Survey 2016. Ministry of Health, Kathmandu, Nepal; 2017.

[59] National Institute of Population Studies (NIPS) [Pakistan], ICF. Pakistan Demographic and Health Survey 2017–18. Islamabad, Pakistan, and Rockville, Maryland, USA: NIPS and ICF; 2019.

[60] Ponna SN, Upadrasta VP, Geddam JB, Dudala SR, Sadasivuni R, Bathina H. Regional variation in utilization of Antenatal care services in the state of Andhra Pradesh. Journal of family medicine and primary care. 2017;6(2):231 10.4103/2249-4863.220024 29302523 PMC5749062

[61] THE WORLD BANK. Low & middle income 2019 [https://data.worldbank.org/income-level/low-and-middle-income.

[62] Arokiasamy P, Gautam A. Neonatal mortality in the empowered action group states of India: trends and determinants. Journal of biosocial science. 2008;40(2):183–201. 10.1017/S0021932007002623 18093346

[63] Farah S, Karim M. Determinants of utilization of antenatal care services in rural area of Bangladesh. Bangladesh Medical Journal. 2015;44(2):67–71.

[64] Ali S, Dero A, Ali S. Factors affecting the utilization of antenatal care among pregnant women: a literature review. J Preg Neonatal Med 2018; 2 (2): 41–45 42 J Preg Neonatal Med 2018 Volume 2 Issue. 2018;2.

[65] Gupta S, Yamada G, Mpembeni R, Frumence G, Callaghan-Koru JA, Stevenson R, et al Factors associated with four or more antenatal care visits and its decline among pregnant women in Tanzania between 1999 and 2010. PLoS One. 2014;9(7). 10.1371/journal.pone.0101893 25036291 PMC4103803

[66] Rai RK, Singh PK, Singh L. Utilization of maternal health care services among married adolescent women: insights from the Nigeria Demographic and Health Survey, 2008. Women’s Health Issues. 2012;22(4):e407–e14. 10.1016/j.whi.2012.05.001 22749200

[67] Beeckman K, Louckx F, Putman K. Content and timing of antenatal care: predisposing, enabling and pregnancy-related determinants of antenatal care trajectories. The European Journal of Public Health. 2013;23(1):67–73. 10.1093/eurpub/cks020 22628457

[68] Chaka EE. Department of Epidemiology and Biostatistics, School of Public Health, Tehran University of Medical Sciences-International Campus (TUMS-IC), Tehran, Iran. Studies.13(15):20–2.

[69] Azimi MW, Yamamoto E, Saw YM, Kariya T, Arab AS, Sadaat SI, et al Factors associated with antenatal care visits in Afghanistan: secondary analysis of Afghanistan Demographic and Health Survey 2015. Nagoya journal of medical science. 2019;81(1):121 10.18999/nagjms.81.1.121 30962661 PMC6433637

[70] Bobo FT, Yesuf EA, Woldie M. Inequities in utilization of reproductive and maternal health services in Ethiopia. International journal for equity in health. 2017;16(1):105 10.1186/s12939-017-0602-2 28629358 PMC5477250

[71] Sarker BK, Rahman M, Rahman T, Hossain J, Reichenbach L, Mitra DK. Reasons for preference of home delivery with traditional birth attendants (TBAs) in rural Bangladesh: a qualitative exploration. PLoS One. 2016;11(1). 10.1371/journal.pone.0146161 26731276 PMC4701391

[72] Mridha MK, Anwar I, Koblinsky M. Public-sector maternal health programmes and services for rural Bangladesh. Journal of health, population, and nutrition. 2009;27(2):124 10.3329/jhpn.v27i2.3326 19489411 PMC2761780

[73] National Institute of Population Research Training, Associates for Community Population Research, ICF International. Bangladesh Health Facility Survey 2014. NIPORT, ACPR, and ICF International Dhaka; 2016.

[74] Mannan M. Access to public health facilities in Bangladesh: a study on facility utilisation and burden of treatment. The Bangladesh Development Studies. 2013:25–80.

[75] World Health Organization. Bangladesh health system review: Manila: WHO Regional Office for the Western Pacific; 2015.

[76] UNDP Bangladesh. Covid-19: A reality check for Bangladesh’s healthcare system 2020 [10/08/2020]. https://www.bd.undp.org/content/bangladesh/en/home/stories/a-reality-check-for-bangladesh-s-healthcare-system.html.

[77] Keya K, Rahman MM, Rob U, Bellows B. Barrier of distance and transportation cost to access maternity services in rural Bangladesh. Population Association of Ameria US. 2013.

[78] Banik BK. Barriers to access in maternal healthcare services in the northern Bangladesh. South East Asia Journal of Public Health. 2016;6(2):23–36.

[79] Walton LM, Brown D. Cultural barriers to maternal health care in rural Bangladesh. Journal of Health Ethics, Fall. 2012.

