# Supplementary material for: Status of the WHO recommended timing and frequency of antenatal care visits in Northern Bangladesh
Source: PLoS One. 2020 Nov 5;15(11):e0241185. doi: 10.1371/journal.pone.0241185 (PMC7644040; doi:10.1371/journal.pone.0241185)
Supplement: S1 File — (DOCX) [file pone.0241185.s001.docx]

**Section-1: Respondent’s identity:**

| **SL** | **Question** | **Responses** | **Code** | **Direction** |
| --- | --- | --- | --- | --- |
| 101 | Respondent ID |  |  |  |
| 102 | Name of Village/Ward |  |  |  |
| 103 | Ward no (current) |  |  | Use code as planned |
| 104 | Name of Union and code |  |  |  |
| 105 | Name of Upazila and code |  |  |  |

**Section-2: Socio-demographic information and Antenatal Care Utilization**

| **SL** | **Question** | **Response** | **Code** | | **Direction** |
| --- | --- | --- | --- | --- | --- |
| 201 | What is your age? | ------ --------  Year Month |  | |  |
| 202 | What is your religion? | 1= Islam  2= Hinduism  3= Christianity  4= Buddhism  5= Other (---------------------------------) |  | |  |
|  |  |  |  | |  |
| 203 | What is your completed year of education? | --------(Completed years of education) |  | | **Write 00 if there is education** |
| 204 | What is your primary occupation/What do you mainly do? | 01= Service (govt.)  02= Service (non govt.)  03= Business  04= Agriculture/Farm/fishing  05= Handicraft  06= Rickshaw puller/Van puller/ boatman  07= Transport worker  08= Skilled labour  09= Student  10= Housewife  11= Unemployed  12= Expatriate  13= Day labour  88= Others(Specify_______________) |  |  |  |
|  |  |  |  | |  |
| 205 | Are you involved in any income generating activities? | 1= Yes  2= No |  | |  |
| 206 | How many times did you become pregnant? (Gravida) | **------------Nos** |  | |  |
| 207 | How many living children you have? | **------------Nos** |  | |  |
| 208 | What is your husband’s completed year of education? | --------(Completed years of education) |  | | **Write 00 if there is education** |
| 209 | What is your husband’s primary occupation? | 01= Service (govt.)  02= Service (non govt.)  03= Business  04= Agriculture/Farm/fishing  05= Handicraft  06= Rickshaw puller/Van puller/ boatman  07= Transport worker  08= Skilled labour  09= Student  10= Unemployed  11= Retired  12= Work in abroad  13= Day labour  88= Others(Specify_______________) |  | |  |
| 210 | Do you watch TV regularly? | 1 = Yes  2 = No |  | |  |

| 211 | | Do you read newspaper or magazine? | | | 1 = Yes  2 = No | |  |  | |
| --- | --- | --- | --- | --- | --- | --- | --- | --- | --- |
| 212 | | Did you receive any antenatal check-up during your last pregnancy? | | | 1 = Yes  2 = No | |  | **If yes, then ask the following questions** | |
| 213 | | If yes, how many times did you have check-ups/ antenatal care during your last pregnancy? | | | **----------------------times** | |  |  | |
| **SL** | **Question** | | **Response** | | | | | | **Direction** |
| 214 | a) Antenatal check-ups | | b) Place | c) Provider | | d) Gestational age of receiving care (Week) | | |  |
|  | ANC-1 | |  |  | |  | | | Use codes for place, providers |
|  | ANC-2 | |  |  | |  | | |  |
|  | ANC-3 | |  |  | |  | | |  |
|  | ANC-4 | |  |  | |  | | |  |
|  | ANC-5 | |  |  | |  | | |  |
|  | ANC-6 | |  |  | |  | | |  |
|  | ANC-7 | |  |  | |  | | |  |
|  | ANC-8 | |  |  | |  | | |  |
|  | ANC-9 | |  |  | |  | | |  |
|  | ANC-10 | |  |  | |  | | |  |
|  | ANC-11 | |  |  | |  | | |  |
|  | ANC-12 | |  |  | |  | | |  |
|  | ANC-13 | |  |  | |  | | |  |
|  | ANC-14 | |  |  | |  | | |  |
|  | ANC-15 | |  |  | |  | | |  |
|  | ANC-16 | |  |  | |  | | |  |
|  | ANC-17 | |  |  | |  | | |  |
|  | ANC-18 | |  |  | |  | | |  |
|  | ANC-19 | |  |  | |  | | |  |
|  | Last ANC | |  |  | |  | | |  |
